# The Development of Stress Reactivity and Regulation in Children and Adolescents

**DOI:** 10.2174/1570159X21666230808120504

**Published:** 2023-08-15

**Authors:** Clarissa Filetti, Finola Kane-Grade, Megan Gunnar

**Affiliations:** 1 Institute of Child Development, University of Minnesota, Minneapolis, USA

**Keywords:** Stress, regulation, development, HPA-axis, children, adolescents

## Abstract

Adversity experienced in early life can have detrimental effects on physical and mental health. One pathway in which these effects occur is through the hypothalamic-pituitary-adrenal (HPA) axis, a key physiological stress-mediating system. In this review, we discuss the theoretical perspectives that guide stress reactivity and regulation research, the anatomy and physiology of the axis, developmental changes in the axis and its regulation, brain systems regulating stress, the role of genetic and epigenetics variation in axis development, sensitive periods in stress system calibration, the social regulation of stress (*i.e*., social buffering), and emerging research areas in the study of stress physiology and development. Understanding the development of stress reactivity and regulation is crucial for uncovering how early adverse experiences influence mental and physical health.

## 1 INTRODUCTION

Adverse early experiences include both the absence of stimulation needed for typical development and the presence of stimuli that threaten healthy development. Early childhood experiences become biologically embedded during development and involve the activity of stress-mediating systems [1]. It is imperative to better understand mechanisms to guide intervention efforts, as early adversity is associated with a range of negative physical and mental health outcomes.

Stress is often defined as a threat, real or implied, to homeostasis, and homeostasis refers to the maintenance of a narrow constriction of vital physiological parameters that are critical for survival [2]. Numerous systems participate in adaptation to stressful stimulation and context; however, the hypothalamic-pituitary-adrenocortical (HPA) axis plays a central stress-mediating role. One of the challenges of studying stress developmentally is that the HPA system and the processes that regulate it and other stress-responsive systems undergo changes with development. Development alters how stressors are experienced, as the systems that perceive and process threat information change with age. Further, the stress-mediating systems undergo change throughout development and are shaped by environmental experiences.

In this review, we discuss what is currently known about the development of reactivity and regulation of the HPA system and the role of experiences, particularly during sensitive periods of development, in shaping how this system responds to stressors and influences downstream health outcomes. As this review encompasses an extensive amount of information relevant to stress reactivity and regulation, it was not feasible to conduct a systematic review for each section. Rather, we began our narrative review by identifying exemplar articles for each section (such as Engel & Gunnar, 2020). Then, using databases such as Google Scholar and PubMed, we expanded our search using pertinent keywords from those articles. Finally, after gathering suitable articles from those searches, we synthesized and summarized the findings in this review. When they were available, greater weight was given to findings based on systematic reviews and meta-analyses.

We begin by reviewing theoretical perspectives that guide research on stress reactivity and regulation and note whether the theory is developmental or not. We continue by discussing the anatomy and physiology of the HPA axis and its development and then outline the brain systems regulating stress and their development. We then review what is currently known about genes and epigenetics in the study of stress reactivity and regulation and consider the role of experience and the possibility of sensitive periods. We follow by examining developmental changes in the social regulation of stress and finish by discussing emerging areas in the study of stress physiology and development, including racial/ethnic inequalities and discrimination and sleep.

## 2 THEORETICAL PERSPECTIVES

Marked individual differences in stress reactivity are a hallmark of stress responding. Environmental and genetic factors have been implicated in individual variation, although the extent to which these components or their interactions contribute to individual differences is actively debated. There are several major theoretical perspectives that guide research on stress reactivity and regulation, only a few of which are clearly developmental. The key theories are described here briefly.

### 2.1 Allostatic Load (AL)

The allostatic load model builds upon the concept of allostasis or the maintenance of stability through the activation of stress systems [3]. Unlike homeostasis, which operates to return systems to baseline after perturbation, allostasis shifts systems to new settings needed to manage the threat to viability. When the threat is brief, stress system responses return to baseline, and other affected systems adjust back to their pre-threat levels. However, when the threat is frequent, prolonged or chronic, the system shifts in functioning can produce maladaptive outcomes (*e.g*., heart disease, chronic inflammation, hippocampal volume reductions), and the stress systems themselves may become dysregulated [4]. This is described as allostatic load or overload. Variation in stress reactivity, and subsequent (mal)adaptations, stem from differentiation in allostatic responses, which are affected by genetics, age, and life experience. The cost incurred from the allostatic load is contextually bound; the interaction between an individual’s biology and behavior informs the encoding of experience, leading to a variety of responses to environmental challenges. The allostatic load model proposes that the prolonged activation of physiological systems to stressful stimuli incurs a cost to the functioning of stress-responsive systems, which affects physical and psychological outcomes. The allostatic load model is not developmental, although it argues that age/time produces an accrual of load which predicts that many diseases of aging reflect a lifetime of stressor exposure [5].

### 2.2 Biological Sensitivity to Context (BSC)

This model is explicitly developmental. Originally formulated by Boyce and Ellis [6], it argues that although traditionally highly reactive stress response systems were thought to be prototypically maladaptive, reactivity may be better conceptualized as enhanced biological sensitivity to context (BSC). The model argues that whether high reactivity results in good or poor health and behavior outcomes depends on whether the developmental context is supportive or adverse. A highly sensitive individual developing in an adverse environment will be more susceptible to the deleterious effects of that environment than will a less reactive individual. The opposite is true for highly supportive environments. Sensitive individuals will reap the beneficial effects more than individuals who are insensitive. Thus, the developmental outcomes depend on the interaction of the quality of the developmental context and biological sensitivity, for better or worse. Variation in BSC is attributed to the interaction of genetic and environmental experiences. Up to this point, the model is highly similar to the Belsky and Pluess [7] differential susceptibility model.

However, drawing on developmental evolutionary (EvoDevo) theory, Boyce and Ellis [6] argue that the developmental context also modifies biological reactivity. That is, the degree of reactivity also reflects a conditional adaptation to early life contexts. Specifically, they argue that in the context of moderate challenges or stressors, reactivity will be decreased, allowing individuals to be less impacted by the stressors they will encounter in their lives. In very supportive contexts, individuals will remain more reactive, which will allow them to reap the full benefits of their supportive environments, but may keep then sensitive to life’s slings and arrows. The focus, though, is more on what happens in very unsupportive, neglectful or abusive contexts of early development. Here the stress system will become even more reactive, leading to enhanced vulnerability to stress-related physical and mental health disorders.

### 2.3 The Adaptive Calibration Model (ACM)

Del Giudice and colleagues [8] embedded the BSC model into a larger theory, the Adaptive Calibration Model (ACM). Using components and themes from developmental plasticity and life history theory, ACM argues that the characteristics of life history, such as rate of growth and learning, sexual maturation and fertility, competition and risk-taking, and pair bonding and caregiving, are regulated by the stress response system (SRS). This system, in turn, is calibrated and recalibrated by life-history relevant information, including the unpredictability/uncontrollability of events, threats and dangers, parental behavior and attachment security, and the morbidity/mortality of those around us. Individual variation in responsivity is the result of feedback from environmental contexts which calibrate the SRS.

Notably, the calibration of the SRS, whether increased or decreased, is considered adaptive, not maladaptive. The SRS and the life-history dimensions its programs have evolved to increase reproductive fitness. The development of stress reactivity and response systems can change in accordance with environmental demands. This ability to change, conditional adaptation, is an evolutionary adaptation. Individual differences within an ACM framework are attributed, although not completely, to conditional adaptation. Therefore, differences in stress reactivity and response are the results of adaptive processes within a context, even if the behavioral or physiological outcomes appear to be maladaptive. The SRS mediates the organism’s development of adaptive life history strategies through the continual feedback of encoded information received from the environment. Drawing upon an evolutionary-developmental biology perspective, the ACM purports that throughout evolution, the human species has experienced both supportive and highly stressful environments, and therefore developmental processes have been shaped by both contexts. Although developmentalists have considered early life stress and adversity to impair development, the ACM takes an adaptationist perspective. The organism’s developmental system will redirect towards strategies that are adaptive within a stressful environmental context, as it would within a supportive environmental context.

Although the ACM proposes that the SRS calibrates and recalibrates at multiple points during childhood and adolescence, data on the HPA axis suggests two primary periods of calibration. One early, during the fetal and infancy period, and one during the peripubertal period. Although this has been demonstrated most clearly in rodent models [9, 10] that have examined the impact of adversity during the perinatal and peripubertal periods, there is also evidence in humans for these two periods of calibration of the axis. In studies of children raised in institutional (orphanage) settings, those removed to family contexts before but not after about two years of age showed patterns of stress responding comparable to children reared in their families from birth, while those placed later showed blunted stress responding [9]. In children reared in institutions and later adopted into families, the HPA axis continued to show blunted responding until puberty [10]. Importantly, this latter study showed that, within individuals, the cortisol response became less blunted as pubertal stages increased over several years.

### 2.4 The Three-Hit Hypothesis of Resilience and Vulnerability

The three-hit model posits that the impact of adversity is a function of hit 1-genetic vulnerability, hit 2-early life environment and hit 3-later life environment [11]. This model builds on and integrates two models not yet discussed, the cumulative risk model and the mismatch model. The cumulative stress model asserts that stressors aggregate over time, and if the sum increases past a particular threshold, the individual is at increased risk for maladaptive mental health outcomes. Conversely, the mismatch model asserts that we have evolved to make biological adaptations to environmental conditions early in life that anticipate the nature of the environment that we will encounter later. If later conditions are similar to early conditions, then we will be more successful. But it not, then these early adaptations will increase the risk of poor outcomes. Exposure to moderately stressful stimuli can promote adaptive responses, a concept known as stress inoculation. The three-hit hypothesis reconciles and adapts these different models of vulnerability and resilience by examining the interaction between genes and the environment.

## 3 ANATOMY AND PHYSIOLOGY OF THE HPA AXIS AND ITS DEVELOPMENT

### 3.1 The Axis

**Fig. (1):**
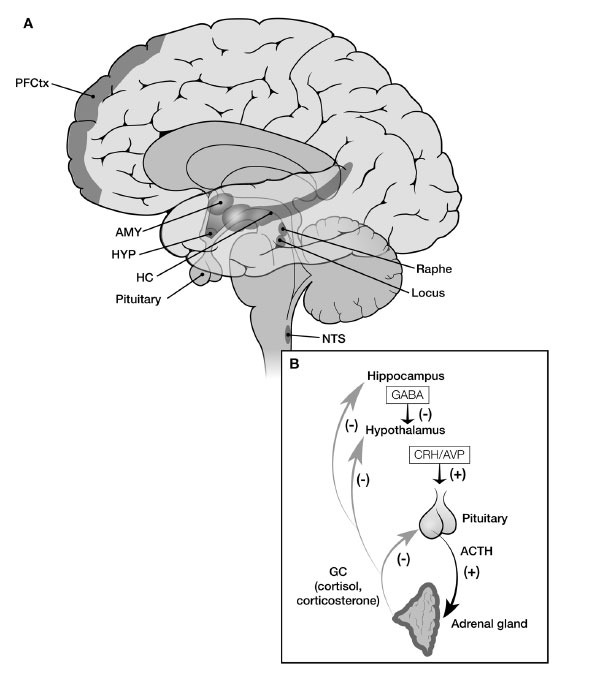
The HPA System. Panel A depicts the anatomy of the HPA system and structures important in its regulation. **Abbreviations:** PFCtx = prefrontal cortex, AMY = amygdala, HYP = hypothalamus, HC = hippocampus, Raphe nucleus (Raphe), Locus coeruleus (Locus), NTS = nucleus of the tractus solitarius. Panel B depicts the activation (+) and negative feedback inhibition (-) pathways of the HPA system. Increases in glucocorticoids (GCs) are initiated by the release of CRH/AVP from the mpPVN in the hypothalamus. Negative feedback inhibition operates through GCs acting at the level of the pituitary, hypothalamus and hippocampus. GCs = glucocorticoids (cortisol in humans), medial parvocellular region of the paraventricular nucleus (mpPVN), CRH = corticotropin releasing hormone, AVP = arginine vasopressin, ACTH = adrenocorticotropic hormone. (Reprinted with permission from Gunnar & Vazquez, 2006).

The head of the HPA axis lies in the paraventricular nucleus (PVN) of the hypothalamus, specifically in the parvocellular neurons in this nucleus (Fig. **1**) [12]. These cells produce the primary releasing hormone of the system, corticotropin-releasing hormone (CRH), as well as arginine vasopressin (AVP) which can amplify activation of the axis. When released, these hormones travel through a short portal system to the anterior pituitary gland. There they stimulate corticotroph cells to produce adrenocorticotropic hormone (ACTH). ACTH is cleaved from a large pro-hormone, proopiomelanocortin (POMC), which also results in the production of other peptides, including ones involved in skin pigmentation and appetite.

ACTH from the pituitary is released into the blood stream and interacts with cells on the adrenal cortex to produce glucocorticoids, cortisol in humans and corticosterone in rodents. The cortex of the adrenal actually consists of three zones with different enzymatic pathways and products. Zona glomerulosa produces mineralocorticoids and is responsive to the salt-water balance of the body as signaled through the renin-angiotensin system. The zona fasciculata is the 2^nd^ layer of the adrenal cortex, and it is this layer that contains the enzymes to convert cholesterol to glucocorticoids. Finally, the third layer, zona reticularis, contains the enzymes to produce androgens, predominantly dehydroepiandrosterone (DHEA) but also androstenedione [13].

### 3.2 Bound *Versus* Free Cortisol

When glucocorticoids are released into the blood stream, like other steroids, they bind to their binding protein, in this case, corticosteroid-binding globulin (CBG, sometimes termed transcortin). The bound hormone is inactive, meaning it cannot enter cells and bind to receptors. Shifts in CBG levels help regulate the availability of glucocorticoids [14]. These different hormone compartments (bound and unbound) are measured differently depending on the matrix (blood, saliva, hair) and assay. Typically, plasma assays, unless modified, yield indices of both the bound and unbound fraction of the hormone. Salivary assays, because only the unbound fraction can pass through the parotid gland, reflect only the unbound fraction and then the subset of that which is not rapidly converted to cortisone by enzymes in saliva. To measure cortisol in hair, the hair is ground into a fine powder and then reconstituted into liquid form and measured using assays that do not differentiate between bound and unbound fractions. Because it is believed that cortisol gets into hair from passive diffusion from plasma or from sweat glands, only the active, unbound fraction is reflected in hair cortisol. However, as in saliva, some fraction of cortisol is converted to cortisone. This conversion is especially active in sweat glands that have high concentrations of type 2 11-β-hydroxysteroid-dehydrogenase (11- β-HSD2) [15]. Thus some have suggested that cortisol and cortisone combined may be a better long-term index of activity of the HPA axis when assayed in hair [16], and that the ratio of cortisol to cortisone might index activity of 11-β-HSD2 [15].

### 3.3 Receptors

The unbound fraction of cortisol (or corticosterone) is lipid soluble and diffuses into all cells of the body. However, to have effects there must be a receptor for the hormone in that cell. Receptors for cortisol typically lie in the cell cytoplasm and once they interact with cortisol or corticosterone, they create a hormone-receptor complex that translocates to the cell nucleus. In the nucleus, cortisol or corticosterone interacts with glucocorticoid receptor elements (GREs) which lie in the promotor regions of many genes. This results in the activation or inhibition of gene transcription. Thus, the primary effects of glucocorticoids are gene-mediated. However, there is also a rapid non-genomic pathway for cortisol to impact neural functioning, as discussed below.

Two types of receptors bind cortisol, mineralocorticoid receptors (MRs) and glucocorticoid receptors (GRs). In most tissues of the body, MRs bind with equal affinity to aldosterone and cortisol and have a higher affinity for cortisol than do GRs. Therefore, at basal levels, MRs are largely bound, while GRs only become bound at stress concentrations of cortisol and at the peak of the circadian rhythm. In aldosterone-target tissues, 11-β-HSD2 converts cortisol to cortisone thus protecting MRs from cortisol [17]. In the brain, MRs are involved in both salt appetite regulation, operating through the nucleus tractus solitarius (NTS) and circumventricular organs, and stress *via* limbic forebrain circuits [18]. In the limbic forebrain, 11-β-HSD2 does not shield MR from glucocorticoids, resulting in the balance between MR and GR, often located within the same neurons, playing a critical role in stress resilience. MR and GR play complementary roles in these circuits to shift energy resources as needed, and alter connectivity to support arousal and defensive behavior and regulate approach/avoidance. MR in the limbic forebrain controls behavioral, autonomic and neuroendocrine responses *via* selective attention, memory, appraisal of threats and selection of coping strategies. MRs in these regions are also involved in encoding experiences, therefore guiding future responses to threats. Because MRs have a higher affinity for glucocorticoids than do GRs, under basal levels, they are mostly occupied and support healthy cell functioning. Under acute threat, MRs in many limbic regions can be rapidly translocated to the cell membrane, where they mediate rapid responses to stress.

GRs are expressed throughout the brain but are in their highest concentration in the CA1 region of the hippocampus [19]. In addition to neurons, GRs are widely expressed in all glia cells, including the myelinating Schwann cells [20]. GR in the prefrontal cortex plays a role in shifting the individual from a reflective to a habitual response mode which supports fight/flight behavior during the threat. GRs are also critically involved in brain plasticity in response to prolong stress [20]. Notably, in different brain regions, the opposition of MR and GR-mediated actions differ. These receptors do mediate the opposing actions of glucocorticoids in the hippocampus, but in the basal amygdala, beta-adrenergic stimulation combined with GR-mediated actions prolongs the rapid cell-membrane MR-mediated response in ways that likely support the encoding of affect-laden information [18]. Chronic hyper-activation of cortisol that results in prolonged occupation of GR (*i.e*., hypercortisolism) and chronic under activity of the system that fails to adequately activate sufficient MR both result in poor physical and mental health [21]. There is increasing evidence that chronic increases in cortisol may upregulate the reactivity of regions like the amygdala while down-regulating PVN CRH-producing cells and/or shifting activation of the axis to AVP [22]. Thus, the blunting of the cortisol stress response often seen in humans after repeated or prolonged activation of the axis may be accompanied by heightened sympathetic activity and central increases in fear and arousal [23].

### 3.4 Timing and Circadian Rhythm

Unlike the sympathetic nervous system, activation of the HPA axis takes minutes to achieve, and its effect can last hours, days or even weeks [24]. A rough estimate is that signals summating to disinhibit CRH neurons in the hypothalamus to peak cortisol levels in plasma takes about 20-25 minutes, with another 2-3 minutes to reach peak concentrations in saliva [25]. The magnitude of response, however, varies with the circadian rhythm, being smaller at the peak of the rhythm and larger near the trough. The circadian rhythm in glucocorticoids typically peaks 30-45 minutes following the transition from sleep to wake (morning in diurnal animals, evening in nocturnal ones) and is at its lowest level 30 minutes after the onset of sleep [26]. In humans, it has been shown that the diurnal rhythm is critical to healthy functioning and is often disturbed under conditions of chronic stress or in relation to disorders such as depression [27]. In addition to circadian rhythmicity, cortisol is produced in pulses, with pulses being approximately 90 minutes apart and varying in amplitude to produce the daily rhythm [28]. As is becoming clearer in studies of individuals on glucocorticoid replacement therapies that do not approximate normal pulsing of the axis, the pulsatile production of cortisol is critical for physical and mental health.

## 4 DEVELOPMENTAL CHANGES IN THE HPA AXIS AND ITS REGULATION

### 4.1 Pre- and Perinatal

There are marked changes in the structure and activity of the HPA axis from conception through the first 6 months or so of postnatal life. Initially, the adrenal cortex consists of a large fetal zone that produces dehydroepiandrosterone (DHEA). This androgen is sent to the placenta, where it is converted to estrogens [29]. Around the end of the first trimester, the zona fasciculata and the zona glomerulosa begin to organize. Although cortisol is briefly produced early in gestation, for the most part, the enzymes necessary for its production are inactive until near the end of the second trimester [29]. Notably, for cortisol to affect the developing fetus, its receptors also must be present. There appears to be complex developmental patterns for MRs and GRs that influence the effects of stress in utero [30].

As the pregnancy progresses, threats to both the mother and the fetus become more likely to result in elevated cortisol, primarily due to CRH stimulation in the placenta [31]. Elevated cortisol in either the mother’s plasma or in the placenta is correlated with lower birth weight, attention regulatory problems, and increased risk of cardiovascular disease. The HPA axis and the maternal-placental-fetal interchange of CRH and cortisol are believed to play an important role in the developmental origins of health and disease DOHaD; [32]. While maternal stress may be transmitted to the fetus and affect its development, cortisol is necessary for the maturation of fetal tissue and the transition to life outside the womb. This may partly explain why unbound levels of maternal cortisol increase in the third trimester, and it is the basis for administering glucocorticoid (typically now betamethasone) to women who may be going into premature labor [31].

### 4.2 Infancy and Early Childhood

The fetal zone involutes following birth and disappear completely by 6 months of life. As it involutes, the three mature zones of the adrenal cortex organize. Despite its structural immaturity, the zona fasciculata is quite capable of producing cortisol at birth and indeed produces marked increases in cortisol during labor and delivery [33]. The liver, however, is immature and does not produce binding globulins, including CBG, at mature levels. Thus, while total cortisol may be low, the unbound fraction of the hormone is quite high in the first months after birth [34]. The HPA axis is highly reactive during the first 3 months postnatal, with stressors as mild as undressing, weighing and measuring producing elevations in cortisol [35]. There is a marked dampening of reactivity to mild stressors between three and four months of age, perhaps due to the development of fast, cell-mediated negative feedback mechanisms [36, 37]. The axis still responds strongly to pain stressors, such as shots or blood draws throughout the first year, although as the attachment system develops, the presence and availability of the attachment figure in a secure relationship provides a powerful buffer of the psychosocial aspects of potentially threatening situations, including receiving one’s inoculation injections [36].

The mature circadian rhythm develops over the first 3 to 5 years of life. At birth, the system shows two peaks that are not aligned with time-of-day [38]. Around 6 weeks, one peak in the morning is observed, and by 3 months of age, an evening nadir, as well as the CAR, can be identified. Napping during the morning and afternoon is associated with a decrease during the nap and a rebound after the nap [39]. As children’s sleep patterns mature, the diurnal cortisol pattern approximates the adult pattern.

Daily cortisol patterns are altered in children who attend full-day child care. Cortisol levels in toddlers and, to a lesser extent, preschoolers rise from morning to afternoon and are higher in the afternoon at child care than they are at home at the same time of day [40]. These elevations are larger in poorer-quality childcare settings [41, 42]. While most children return to expected low levels of cortisol when they go home at night [43], daily elevations at child care predict increased behavioral problems among children who are temperamentally shy and anxious [44]. The longer-term impact of attending childcare, though, may depend on the family risk status of the child. Among low-risk children, child care attendance predicts increases in cortisol over time, while the opposite appears to be true for children from high-risk family situations [45].

### 4.3 Childhood and Adolescence

The adrenal cortex plays an important role in pubertal development [46]. The zona reticularis produces androgens; most notably, for puberty, it produces DHEA. This hormone which was so important in sustaining a pregnancy, initiates the early phases of puberty, the phase known as adrenarche. Adrenarche begins between 5 and 8 years in girls and 7 and 11 years in boys, and last until about age 20. While external indicators of adrenarche are typically not seen for several years after DHEA concentrations begin to rise, as DHEA increases, this hormone participates in the production of pubic and body hair, increased oily skin and acne, and changes in sweat composition that then produces body odor. ACTH is not involved in adrenarche; however, ACTH does stimulate the production of DHEA along with cortisol in response to stressors. DHEA, an anabolic steroid, and cortisol, a catabolic steroid, have opposing effects on many organs and tissues. Thus, the increase in DHEA with adrenarche and its production in response to stressors may serve some protective functions.

Central puberty involves the awakening of the hypothalamic-pituitary-gonadal axis, which in girls ultimately leads to cycling and ovulation [47]. Notably, cortisol levels also increase with central puberty, peaking around ages 10-13 or Tanner stage 3 [13]. Some suggest that cortisol stress responses also increase with puberty [48], but this may actually reflect increased sensitivity to social evaluation, the most effective stressor in most laboratory stressor tasks [49]. Sex differences in HPA reactivity and regulation develop with puberty, including variations in responses to psychosocial stressors with the menstrual cycle [50].

## 5 BRAIN SYSTEMS REGULATING STRESS AND THEIR DEVELOPMENT

**Fig. (2):**
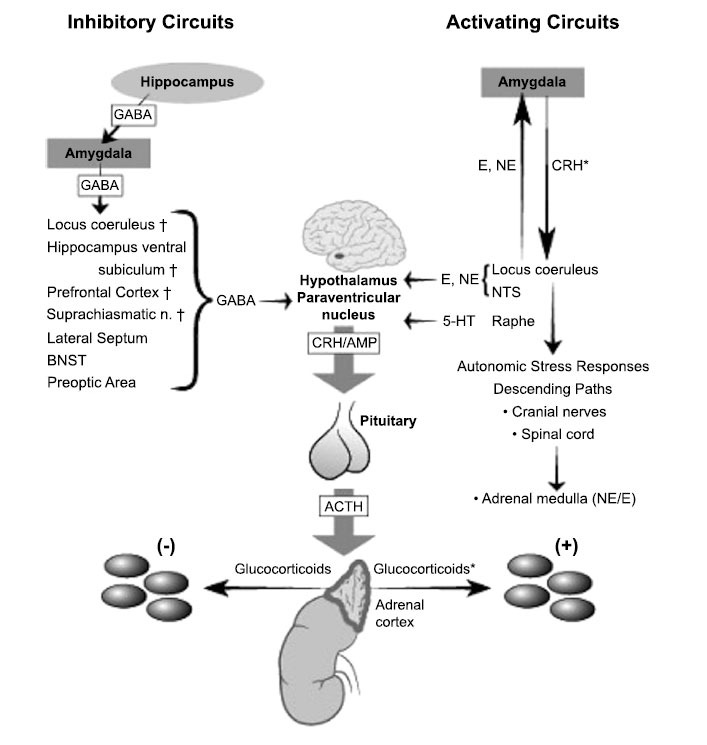
Schematic representation of the activating (right side) and inhibiting (left side) circuits that contribute to regulation of the HPA system. (Reprinted with permission from Gunnar & Vazquez, 2006 [13]).

In addition to changes in the HPA axis itself with development, the brain systems that activate and regulate the axis also undergo normative developmental changes and are affected by early life stress in ways that affect axis reactivity and regulation. Here we outline the major activating and regulating neural systems influencing axis activity. Both ascending information about the body’s state and descending information about the perception of threat activate the HPA axis. Whether the axis is activated, how strongly and how long is the result of a summation of information coming from bottom-up and top-down activation and inhibition pathways (Fig. **2**).

### 5.1 Bottom-Up and Local Regulation

#### 5.1.1 Brain Stem

The HPA axis responds to bodily signals that indicate deviations from homeostasis. These activating pathways operate without higher cognitive processing. The stimuli that activate this pathway are sometimes termed systemic stressors. Brain stem catecholaminergic centers have an integral role in regulating the HPA axis [51]. There is an excitatory role of brainstem nuclei on ACTH and glucocorticoid release due to ascending information signaling systemic deviations from homeostasis [52] and possible transmission of descending signals from limbic forebrain structures [12]. Prior research has ascertained that the nucleus tractus solitarius (NTS) is the main mediator of the majority of these HPA axes excitatory responses [53]. The NTS is well-developed at birth and is located in the dorsal medulla in the hindbrain.

#### 5.1.2 Hypothalamus

In addition to activation and negative feedback regulation within the HPA axis, there is evidence that the drive of the axis may also be inhibited by PVN-projecting hypothalamic neurons. Most of these are GABA-ergic and thus inhibitory [54]. There are also nuclei within the hypothalamus that modulate inhibition of PVN-CRH neurons, including the dorsomedial hypothalamic and posterior hypothalamic nuclei and the nuclei of the medial preoptic area. Some of these provide input that increases and some input that decreases CRH production. These nuclei receive input from the prefrontal cortex and thus may be relay stations for top-down regulation.

### 5.2 Top-down Regulation by Cortical and Limbic Regions

Top-down regulation of the HPA axis does not act through a single brain area; rather, several brain areas and neurotransmitters are involved in activating and inhibiting stress responses. Glucocorticoid receptors (GRs) are widely distributed in the brain, with the highest numbers found in the amygdala, hippocampus, and medial prefrontal cortex [12], regions also frequently implicated in psychopathology. However, other regions of the prefrontal cortex also contain GRs and are sensitive to chronic elevations in cortisol.

#### 5.2.1 Amygdala

In response to psychological (sometimes called processive) stressors, the HPA axis appears to be activated by input from the amygdala, mediated primarily by the medial (MeA) and central (CeA) nuclei [12]. As the majority of MeA and CeA projections are GABAergic, activation of the axis by these structures is thought to be mediated by disinhibition (*i.e*., blocking of tonic PVN inhibition through GABAergic projections). The MeA receives olfactory input and is critical in the control of social behavior and aggression in rodents [55,56]. The CeA receives signals from the thoracic and abdominal viscera and is a critical regulator of autonomic nervous system responses [57]. Both the CeA and MeA impact the PVN through projections to the bed nucleus of the stria terminalis (BNST), and projections from the BNST to the PVN are GABAergic. Further, both regions receive excitatory input from the basolateral amygdala BLA; [58]. When cortisol binds to glucocorticoid receptors (GR) within the amygdala, it leads to a feed-forward input to the HPA axis, which prolongs the duration of the stress response [59]. Therefore, the amygdala plays an important role in both the activation and duration of the HPA axis stress response.

Rodent studies suggest that a dysregulated stress response produces alterations in the structure of the amygdala and hippocampus [60]. However, the direction of impact is not clear. While some researchers have found that elevations in corticosterone are associated with larger amygdala volumes [61], others have noted the opposite effect [62, 63]. There is more consistent evidence that glucocorticoids increase amygdala reactivity and, conversely, reduce prefrontal cortex regulation of the amygdala. These effects would shift the individual from more reflective and analytic cognitive states (*i.e*., higher executive functioning) to reflexive, defensive cognitions and actions [64].

Consistent with its role in emotion and emotional learning, the primate amygdala is relatively mature at birth, with little evidence of the addition of new neurons after about 3 months of age. Nonetheless, it undergoes a prolonged period of developing connectivity with other structures. Stereological analysis of the macaque monkey has revealed a rapid increase in volume in the lateral and basal amygdala between birth and 3 months of age (*e.g*., birth to a year in human development), followed by slower growth until about one year (*e.g*., 4 years in humans) [65]. MRI studies in humans have also noted a period of marked volumetric increase in the amygdala between 8 years and 18 years of age [66]. These increases are due to increases in oligodendrocytes, likely reflecting increases in connectivity within the amygdala and with extra-amygdala structures, most notably those in the prefrontal cortex [33]. Interestingly, there is a region in the primate amygdala adjacent to the basolateral amygdala called the paralaminar nucleus. The neurons in this area are immature at birth and gradually develop. These neurons may be especially sensitive to early life stress; indeed, early life stress in the form of maternal loss in the first months of life has been shown to alter gene expression in this region later in development in ways consistent with the altered social learning in animals with a history of maternal loss [67]. From a regulatory standpoint, the medial prefrontal cortex is important in inhibiting amygdala responses to threatening stimuli. Medial PFC to amygdala connectivity, however, tends to be positive early in life, shifting to negative in adolescence; however, the shift from excitation to inhibition appears to occur earlier for individuals who lack a supportive caregiver early in life [68]. This finding is consistent with other evidence of early life stress advancing maturation of neural circuits needed for independence.

#### 5.2.2 Hippocampus

The hippocampus is integral in regulating the circadian rhythm and responses to psychogenic stressors. As noted, the hippocampus plays an important role in genome-mediated feedback inhibition of the HPA axis [69]. This is supported by the expression of GRs and mineralocorticoid receptors (MRs) in hippocampal neurons [70], with GRs mostly involved in negative feedback regulation of the axis. The hippocampus exerts a largely inhibitory influence on the PVN [53]. Lesion studies in rodents indicate that extensive hippocampal damage of the dorsal and ventral hippocampus increases PVN CRH mRNA expression and enhances corticosterone responses to stress [69]; however, that does not always hold true [71].

The ventral hippocampus is integral in the inhibition of the HPA axis *via* communication with ventral subiculum (SUBv) cortical output neurons. Neurons in this region project to several subcortical regions that in turn project to the PVN, including the bed nucleus of the stria terminalis (BNST), medial preoptic area, and dorsomedial and poster hypothalamic nuclei [72-74]. The majority of PVN inputs from the preoptic area and BNST are GABAergic, inhibiting the HPA axis [72].

Research has largely established a relationship between a smaller hippocampal volume and elevations in cortisol, but some have posited that this relationship is only present for certain regions of the hippocampus [75, 76]. On a circuit level, we do not yet know when hippocampal regulation of the PVN begins in development. The SUBv and several other hippocampal regions are relatively mature at birth [77]. Glucocorticoids have an integral role in the maturation of the hippocampus.

#### 5.2.3 Prefrontal Cortex

Two regions in the rodent have been studied extensively with regard to the regulation of the HPA axis. These regions are the prelimbic cortex (PL) which is broadly homologous with the dorsal anterior cingulate cortex (dACC) in humans, and the infralimbic (IL) cortex which is considered roughly comparable to Broadman’s area 25 (BA25) in humans. From lesion studies in rodents, we have learned that the infralimbic (IL) and prelimbic (PL) divisions of the medial prefrontal cortex (mPFC) are differentially involved in inhibiting the HPA axis in response to stressors. These regions respond to psychological rather than physical/systemic stressors, as inactivation of the IL/PL and PL or damage to those regions increase corticosterone and ACTH responses to novelty but not too low levels of oxygen in body tissues [78, 79]; however, in response to repeated homotypic stress, damage to the right IL/PL reduces corticosterone secretion [80]. Whether these processes are analogous in humans is not known.

From research with humans, we have learned that the medial prefrontal cortex (mPFC) plays an integral role in processing threats, and its activity increases in response to stressors [53]. The mPFC receives projections from other limbic regions and restrains the activity of the HPA axis. When in development, the mPFC regulation of the HPA axis begins is also unknown. mPFC inhibition of the HPA axis operates *via* connections through the NTS, the BNST (often considered the extended amygdala) and the amygdala. mPFC inhibitory input to the amygdala develops over childhood and into adolescence [81]. Interestingly, there is evidence that when the attachment figure is present, mPFC to amygdala inhibition is increased in childhood in a way that presumably would support more mature emotion regulation and stress-buffering [82].

### 5.3 Chronic Stress

In adult animal, chronic stress alters pathways delivering information about the threat to the hypothalamic PVN. Notably, the posterior paraventricular nucleus of the thalamus (PVT) is selectively involved in the control of HPA axis responses to chronic stress rather than acute stress. The PVT projects to the amygdala and prefrontal regions involved in HPA axis regulation and receives input from these regions [83]. Lesions of the PVT block sensitization of the HPA axis by chronic cold exposure [84]. Further, habituation of HPA axis responses can be blocked by chronic inhibition of GRs and MRs in the PVT, which points to glucocorticoid signals being essential to this process [85]. Pathways altered under chronic stress in mature animals include circuits in the ventral hippocampus and ventromedial prefrontal cortex. Hypothalamic CRH neurons in the PVN are under inhibitory (GABAergic) control, so excitatory inputs to PVN GABAergic cells increase the inhibition of CRH, which leads to reduced CRH drive [86]. Chronic elevations in cortisol can have positive feedback impacts on the central nucleus of the amygdala, increasing the volume of the amygdala and increasing CRH, thus increasing reactivity to future stressors [86]. Chronic CRH impact on the pituitary gland can be accompanied by the downregulation of pituitary ACTH production, reducing the capacity of CRH to stimulate cortisol production by the adrenal gland [87]. However, chronic activation of the adrenal cortex increases its volume and makes it more sensitive to ACTH stimulation [87]. Therefore, the impacts of chronic stress are complicated, and both hyper- and hypoactivity of the HPA axis can develop.

### 5.4 Chronic Stress During Development

Early childhood is a period of rapid neurobiological growth; thus, chronic stressors during development can have marked impacts on the developing brain [88]. In rodents, chronic stress in the form of reduced or unpredictable maternal care increases the reactivity of the HPA axis, in part through effects on DNA methylation that impact GR gene expression in hippocampal and prefrontal regions important for inhibition of the axis in response to psychosocial stressors [89]. Notably, in monkeys and humans, chronic stress during development tends to be associated with hypocortisolism [90, 91]. Rodent studies suggest that a dysregulated corticosterone stress response produces alterations in the structure of the amygdala and hippocampus [60]. It is possible that early changes to the structure of the hippocampus and amygdala could disrupt their critical functions and, in doing so, impair emotional and cognitive development.

Children who were previously institutionalized (PI) have been shown to have reduced hippocampal volume and reduced growth rate of the amygdala, resulting in smaller volumes by adolescence [92]. Both hypo- and hypercortisolism have been found to be associated with heightened psychiatric symptoms in children [93, 94]. Likewise, alterations in the hippocampus [95] and amygdala [96] have also been found in children with psychiatric symptoms. Therefore, the relationship between cortisol stress response and brain structure as children develop may contribute to the development of psychopathology. Timing is also critical, as rodent studies suggest that the developing brain is more susceptible to stress than the mature brain [97].

## 6 GENES AND EPIGENETICS

Understanding individual variation is central to the study of stress reactivity and regulation. While much research has been devoted to identifying the psychosocial processes and experiences underlying these differences, it is widely acknowledged that genetic variation also plays a role. As in other areas of psychology, the expectation is that genetic differences may both create vulnerability to dysregulation of the stress system in response to stressors *i.e*., stress-diathesis models; [98] and increase the individual’s sensitivity to experience in a for-better-or-for-worse manner *i.e*., differential susceptibility models; [7]. Examinations of the heritability of HPA axis activity have shown high heritability for morning levels of cortisol both in children and adults [99]. A recent study of 8- to 15-year-old twins, however, revealed that distinct genetic variation was associated with the response to stress, in this case, the Trier Social Stress Test, compared to results for diurnal measures of cortisol and hair cortisol [100]. Such behavior genetic findings suggest that different genotypes underlie different aspects of HPA functioning.

Given this, it is not surprising that over 30 genes have been identified with functional mutations and variants that influence glucocorticoid synthesis, metabolism and HPA axis regulation. These genes are listed in Table **S1** in Supplemental Materials. Some of these genes have been the focus of single-gene studies, while others have been used to create polygenic risk scores. Most of the work examining genetic polymorphisms and DNA methylation of genes involved in the HPA axis has not focused on the regulation of the axis but on physical and mental health disorders that may be influenced by these genes [101]. Here we focus on the activity of the axis as the endpoint. While single genes rarely account for significant variance in behavioral outcomes, those involved in critical steps in the regulation of glucocorticoid production may have significant impacts on the stress system. Here we highlight two genes that have been of singular interest.

### 6.1 Candidate Gene Studies of FKBP5 and NR3C1

The *FKBP5* gene produces a binding protein that, among its other molecular activity, acts as a co-chaperone binding to heat-shock protein 90 and other co-chaperones of the GR receptor to inhibit GR signaling [102]. Through modulating GR signaling, FKBP5 can impact the actions of cortisol with effects on every body tissue. There is a dynamic pattern of relations between increases in cortisol (*e.g*., stress response) and the transcription of the FKBP5 gene. As described earlier, when cortisol increases in response to a stressor, GR is bound and translocates to the cell nucleus, where it interacts with GREs to modulate gene transcription. There are GREs upstream of the FKBP5 promotor that enhance the transcription of the binding protein. Thus, with stress, cortisol elevates, and GR interacts with the FKBP5 gene to increase the production of FKBP5, which then serves to reduce the sensitivity of GRs to cortisol, thus dampening cortisol’s effects, including reducing the negative feedback mechanism mediated by GR.

There are numerous single nucleotide polymorphisms (SNPs) in the FKBP5 gene, the most studied ones comprising a haplotype that spans the whole gene. This haplotype is associated with larger increases in FKBP5 in reaction to GR activity [103]. Further analyses of these SNPs have identified one of the 18 in this haplotype (rs1360780) as the functional variant. Approximately 35% of the white population carries the version of this gene that confers large increases in FKBP5 in response to GR activity. Increases in the expression of FKBP5 in the amygdala and hippocampus are associated with heightened anxiety and slower extinction of conditioned fear responses [102]. Thus, this allele increases the risk of prolonged stress-induced anxiety and fearfulness. This genetic polymorphism has been associated with a variety of stress-related psychological disorders, most notably post-traumatic stress disorder.

The FKBP5 gene also has been shown to be a target of epigenetic changes in response to adverse experiences, especially those occurring early in life. Changes to the epigenome involve a number of biochemical processes; however, DNA methylation produces the most long-lasting changes in response to early life adversity [104]. Notably, there is considerable evidence that methylation is allele-specific, which likely means that the genetic context moderates epigenetic changes induced by stressors. The FKBP5 gene has been shown to undergo DNA methylation alterations in response to trauma and adversity. Specifically, reduced DNA methylation has been shown in FKBP5 intron 7 GRE that then potentiates FKBP5 production in response to GR activation [102]. Importantly, there is little evidence that this epigenetic change occurs in response to traumas experienced in adulthood, but increasing evidence that traumatic experiences early in life produce this change. Of particular note, trauma has larger epigenetic impacts on the FKBP5 gene for individuals carrying the risk version of the gene, but only during early development [105]. These types of results indicate that there are periods in development when cells are particularly sensitive to FKBP5 epigenetic effects. Critically, however, like all epigenetic effects, these are likely to be tissue-specific, and how they impact stress reactivity and coping are also likely to be dependent on the brain regions affected. A challenge to human research is that we cannot directly access the brain tissue of interest but must rely on changes in the periphery (*e.g*., saliva, blood, buccal cells).

The *NR3C1* gene carries the code for the receptor that binds cortisol in the periphery of the body and that, in the brain, is bound when cortisol increases during stress. GRs are widely distributed in the brain and periphery. While there are polymorphisms in this gene that increase and decrease sensitivity to cortisol and have been associated with physical health problems [106], studies focusing on this single gene alone are more often examining experience-induced epigenetic changes. This focus emerged from research on the effects of early handling on HPA axis reactivity and regulation [107]. This effect was later traced to increased GR expression in the hippocampus and prefrontal cortex [108]. Still, later, it became evident that epigenetic modifications of the GR gene were produced in the postnatal period in rats by variations in maternal care induced in rat dams by disturbing their nest and handling their pups [109]. Variations in the quality of parental care occur normally in most altricial species, including our own, but can be increased through perturbations that impact the mothering figure.

Because DNA methylation is tissue-specific, translating the animal work to human development would ideally involve the measurement of methylation of the GR gene in brain tissues. One study did so by examining postmortem hippocampi obtained from individuals who committed suicide and who did or did not have histories of early trauma and abuse. Results were somewhat consistent with the rodent work [110]. The other studies have examined GR methylation in immune cells in blood samples and the variety of saliva and buccal cells. Saliva and buccal cell sampling are the two most common DNA in epigenetic studies of children [111]. Six studies with children have been conducted examining NRC31 methylation, and most support the hypothesis that higher childhood adversity is associated with higher methylation of this gene. In studies of adults with childhood maltreatment histories, 20 studies were retrieved in a systematic review by Parade *et al.* [111], and while the majority noted positive associations of NR3C1 methylation with child abuse, there were also considerable variations in findings which may relate to measures of child abuse (often retrospective self-reports which are unreliable) and the inclusion of adults with psychiatric disorders and medication use.

### 6.2 Genome-wide Association Studies (GWAS) and Polygenic Risk Scores (PRS)

GWAS studies have been used to identify single gene polymorphisms (SNPs) associated with individual differences in various aspects of HPA axis functioning (*e.g*., plasma levels of cortisol, measures of binding globulin, cortisol awakening response (CAR), response to dexamethasone (DEX) suppression, diurnal rhythm). For example, Gerritsen and colleagues [112] conducted a large GWAS study examining targeted genes known to be involved in different aspects of HPA axis functioning. They examined both the CAR and DEX suppression using a very large sample of Northern Europeans. None of the 30 genes and over three thousand SNPs on these genes was significant after false discovery rate (FDR) correction, although there was a borderline significant finding for the SERPINA6 gene that codes for cortisol binding globulin (CBG). For DEX suppression, both the LEP, which codes for Leptin, and AKR1D1, which codes for the 5β-reductase that inactivates cortisol in the liver, were identified.

The Gerritsen and colleagues [112] finding for the SERPINA6 gene, although only marginally statistically significant, replicated an earlier finding by the CORtisol NETwork (CORNET) consortium [113]. In addition, CORNET recently doubled its sample size to over 25 thousand individuals and verified the original SERPINA6 finding. Notably, in the extended sample, they also showed that this gene that encodes for CBG also affects the delivery of cortisol to peripheral tissues and increases the risk for ischemic heart disease [114].

The Gerritsen and colleagues [112] paper also examined the interactions of their GWAS findings with childhood maltreatment. Here they found that the LEPR (leptin receptor) gene interacted with child maltreatment to predict the CAR, while the gene coding for MR interacted with child maltreatment to predict the response on the DEX suppression test. These researchers were also interested in HPA axis-associated genes that predicted hippocampal and amygdala volume as well as major depression. Interestingly, in interaction with child maltreatment, the gene coding for MR predicted all of these phenotypes. An earlier study, however, found that two SNPs on the FKBP5 gene were associated with the total output of cortisol over the day in both a large sample of adults in the Rotterdam Study and in the Whitehall study. None of the other 33 genes with thousands of SNPS survived FDR and replicated on the Whitehall cohort [115].

Using a PRS score based on the Bolton study described above, a recent study of adolescents did not find any association with the risk score based on the GWAS for plasma cortisol in predicting hair cortisol concentrations [116]. However, a number of studies have now used a polygenic risk score based on 10 SNP located on the CRH receptor 1 gene, the MR gene, and the FKBP5 gene [117]. This PRS has been shown to interact with life stress among children to predict higher cortisol reactivity and a less marked diurnal cortisol slope, as well as hippocampal and amygdala volume and connectivity [117]. A recent publication showed that the HPA PRS increased the risk of depression for adolescents who experienced childhood adversity and current stressful events [118]. There is also increasing evidence that PRS scores based on SNPs in the SERPINA6 gene, as well as some loci in the SEPRINA2 and SERPINA1 gene influence diurnal cortisol and the stress response. In a study of 8-year-old Finnish children, a PRS score based on data from the CORNET consortium showed that children with higher PRS score has a smaller decrease in cortisol over the day and mounted a larger response to the Trier Social Stress Test [119]. Interestingly, in that study those children below the median on the PRS score did not show any cortisol response to the stressor task. In that study, single SNP analyses yielded significance for one SNP in SERPINA6 and one in SERPINA1, which combined appeared to carry the effects of the PRS.

Interestingly, in a recent genome-wide DNA methylation study, no CpG loci in HPA-related genes were identified as mediating associations between childhood trauma and adult cortisol reactivity to stressors. Instead, loci in KITLG, the ligand for receptor-type protein-tyrosine kinase, which is critically involved in cell proliferation and survival, was found to mediate between childhood stressors and the lower cortisol response to the Trier Social Stress Test noted for individuals reporting more childhood trauma [120]. This paper involved three independent replication samples, with one being made up of youths 15-18 years. The paper also involved an examination of brain-cortisol associations from deceased individuals which indicated that expression of KITLG in regions of the prefrontal cortex correlated with plasma cortisol levels obtained earlier, prior to death. As the authors note, the involvement of the KITLG protein in HPA axis stress reactivity is biologically plausible because the levels of KITLG correlate with GR expression in response to stress-induced red blood cell production. Not surprisingly, genetic regulation of the HPA axis extends beyond genes involved directly in axis activity. However, a discussion of these genetic findings is beyond the scope of this review.

## 7 SENSITIVE PERIODS

The idea that there are sensitive periods when the stress system calibrates key regulatory processes is central to several of the theoretical perspectives on stress outlined earlier. Sensitive periods and their mechanisms have been well-established for sensory systems, as experienced-expected sensitive or critical periods of circuit refinement build up to allow complex functions, such as language, to emerge from more primitive competencies [121]. Sensitive periods and their mechanisms are difficult to study in humans because of the lack of experimental control over the onset and offset of stimulus inputs and the typically invasive methods needed to identify mechanisms. This is especially the case for examining sensitive periods in the organization of stress reactivity and regulation. Presumably, these periods are ones in which stress systems become calibrated to the degree of stress and challenge in the environment (*e.g*., Del Giudice *et al*. [8] 2011). Thus, establishing sensitive periods would mean having experimental control over the harsh *versus* supportive experiences children have at different points in their development. As will be discussed, there are some rare examples of experimental control over removal from harsh to more benign conditions, but for the most part, our understanding of sensitive periods is based on animal work and some natural experiments. Studies in rats and mice indicate a sensitive period in the first few weeks after birth when the glucocorticoid receptor gene’s DNA methylation status in the forebrain is shaped by maternal care [104]. Then there is another sensitive period during pubertal development whose mechanisms might be related to elevated concentrations of ACTH in the pituitary and glucocorticoid levels in the adrenals, despite similar circulating levels [122].

### 7.1 The Prenatal Period

The Developmental Origins of Health and Disease (DoHAD) model argues that critical set points for many systems are established during fetal development based on signals that arrive *via* the placenta to influence the fetus and prepare it for the nature of life outside the womb [123-125]. Intrauterine stress affects the development of the HPA system and reduces the size of the body, including the nephrons, elevating the risk for high blood pressure and cardiovascular disease as the individual ages [126]. While Carpenter and colleagues found that low birthweight, a proxy for prenatal stress, was associated with both hyper- and hypocortisolism in adulthood, a number of other studies have found that it is associated with higher fasting plasma cortisol and increased reactivity of the HPA axis to psychosocial stressors [127], although this effect may be specific to females. Greater sensitivity of female fetuses to maternal stress might reflect a greater permeability of the female placenta to maternal cortisol due to a larger decrease in 11β-HSD enzymes in response to stress in females as compared to male placentas [128]. This sex difference likely has a protective effect on the viability of carrying the baby to term. According to the viability-vulnerability hypothesis [129], female fetuses slow their growth more in response to a harsh intrauterine environment which increases their chance of surviving difficult gestations in which nutritional resources are scarce.

There are likely multiple pathways through which maternal stress during pregnancy impacts fetal development and later stress regulation and health. First, elevated levels of glucocorticoids in the fetus negatively impact the developing hippocampus, as has been demonstrated in Rhesus macaques [130]. Maternal stress and inflammation have multiple impacts on fetal development [131]. Poor maternal nutrition, especially undernutrition, leads to elevated activity of the HPA axis and reductions in GR expression. In turn, this results in reduced 11β-HSD2 levels in the placenta, which permits more maternal cortisol to cross into the fetal compartment. The elevated levels of cortisol in the fetus increase the production of fetal fat cells, which produce inflammation and program the later development of metabolic syndrome [132]. Maternal use of drugs and alcohol may also alter HPA axis functioning. A meta-analysis of studies conducted through 2012 on children under the age of 5 years reported that, of the teratogens (*e.g*., smoking, drug use, alcohol) studied, maternal alcohol consumption had the largest effect size on elevated cortisol baseline [133]. However, a recent study of preschoolers found that maternal alcohol consumption measured in meconium collected at birth was associated with lower (not higher) cortisol measures in both saliva and hair [134].

As this last study suggests, prenatal stress is sometimes associated with hypoactivity of the HPA axis, not hyperactivity. Indeed, a meta-analysis found about equal effect sizes for both hypo- and hypercortisolism [133]. Similar results have been obtained for the effect of chronic postnatal stress *e.g*., [27]. What accounts for these two patterns in the context of prenatal stress is unclear, but these patterns might reflect the timing of the stressors. Several mechanisms regulate the impact of maternal stress on the fetus and these change during gestation. For example, the expression of 11β-HSD2, the enzyme that converts cortisol to cortisone, making it inert, changes during gestation. It is high early in gestation, thus protecting the fetus from maternal cortisol, but is lower near the end of gestation, allowing maternal cortisol to affect the fetus, presumably as part of maturing the fetal lungs and other tissues in preparation for delivery [29, 33, 135].

### 7.2 The Early Postnatal Period/Infancy

Animal models, particularly non-human primate models, are useful in considering postnatal sensitive periods for stress regulation. Non-human primates are born with a maturity more like humans than rats and mice. And in studies with non-human primates, the timing of postnatal events can be more accurately determined than in studies of humans. Furthermore, cross-fostering designs in these primate studies allow the effects of experience to be differentiated from genetics. In studies of Rhesus monkeys, early life stress (ELS) in the form of maltreatment in the first 3 months of life produced elevated cortisol, which then also in adolescence predicted a decrease in white matter integrity in the corpus callosum, among other brain regions [136]. In these same animals, as juveniles, both left and right amygdala volumes were positively associated with how much abuse the animals suffered as infants [137]. Notably, in this model, the mothers spontaneously abuse their offspring, and they do so with each offspring they birth. This allows a cross-fostering design as the females who abuse are known before delivery. Also, the first 3 months are roughly equivalent to the first year in human development. Thus, these data suggest that infancy is a critical postnatal period for calibrating the HPA axis.

In humans, evidence generally suggests that early maltreatment is associated with smaller hippocampal volume and larger amygdala volume later in life [138]. But it is not clear if there is a sensitive period for these effects. Kuhlman and colleagues [139] attempted to examine the timing of maltreatment and reported findings suggesting that traumatic exposures prior to the first birthday were predictive of prolonged responding (*i.e*., slower recovery) from a stress task. This might implicate altered negative feedback regulation. One of the challenges of studying the impact of maltreatment is that it is predictive of psychopathology, which may be both a reflection of altered stress regulation and an influence on stress system functioning [1]. For example, in a study of school-aged children, those who were maltreated before age 5 showed altered cortisol production over a day at summer camp, but this was only true for those children who also exhibited elevated internalizing symptoms [140]. Of course, this would be consistent with the three-hit models, arguing that genetic vulnerability, plus early life stress, are needed to observe altered HPA regulation. In sum, while the maltreatment literature suggests that the HPA axis is implicated, it has yet to provide substantial evidence for sensitive period effects.

One interesting way that sensitive periods be examined in human development is to investigate the timing of maternal depression (*i.e*., active *vs*. in remission) on the offspring of depressed women. In several studies, active depression when the baby is under a year of age has been associated with long-term impacts on the axis [141]; nonetheless, other studies have suggested that the effects are cumulative or later periods might be more important [142]. One challenge in using the literature on maternal depression to try to identify sensitive periods is that families with more resources may be able to partially buffer the child during maternal depressive episodes. Additionally, even during periods of remission, because of the relationship’s history, the mother-child interactions may continue to be stressful.

Some of the best evidence for early postnatal sensitive periods comes from studies of children reared in institutions (*e.g*., orphanages) and then adopted or fostered into supportive homes. Institutional care, especially care of infants and toddlers, typically results in significant delays in cognitive, motor, emotional and language development [143]. There is also evidence of aberrant diurnal cortisol rhythms for children living in institutional settings [144, 145]. Once removed from institutional care and placed in supportive families, children rebound, showing remarkable improvements in both physical and mental development [146]. Nonetheless, depending on how long they are in institutional care and how severely depriving that care was, effects on the HPA axis can be observed for years following removal. Two studies following children adopted after 6 to 8 months from severe and global deprivation in the Romanian institutions of the early 1990s, showed long-term impacts on the axis [147, 148]. Since children in Romanian institutions of that era experienced incredibly harsh conditions, it is rather amazing that if adopted by 6 to 8 months, the axis was plastic enough so that no long-term impacts were noted. When conditions are less harsh, as indexed by less severe growth restriction and, in one case, direct observation, the period of great plasticity seems to extend to the middle or end of the 2^nd^ year of life. Thus, Leneman *et al*. [149] observed that children adopted after about 1.5 years of age showed a blunted cortisol awakening response, while those adopted earlier showed a response similar to children growing up in homes comparable in socioeconomic class to those of the adopted children. More substantially, in the one study of random assignment to care-as-usual *versus* removal from institutions and placement in foster families, in middle childhood children placed in families by 2 years of age responded to a stressor task similar to children born and raised in their families; while children who were placed later and those in the care-as-usual group showed a marked blunting of the cortisol stress response [9]. This last study is critically important not only because of the random assignment to foster care *versus* care-as-usual but because, in animal studies, it is the reactivity of the axis, not the basal levels, that are shaped by early adversity.

To our knowledge, other studies of the diurnal rhythm in previously-institutionalized youth have not addressed the question of sensitive periods [150]. Thus, there is some suggestion that, at least with regards to institutional deprivation, the sensitive postnatal period may close sometime between about 1.5 and 2 years. More studies are needed, however, to be sure this is the case.

### 7.3 Pubertal Recalibration/Adolescence

As noted in the section on theories, the Adaptive Calibration Model [8] argues that there are multiple periods of calibration and recalibration across childhood. However, with regards to the HPA axis, there is reason to suspect that calibration occurs during a prenatal/infancy period, and then another sensitive period during pubertal development allows recalibration of the system. Puberty is a period of heightened plasticity in many systems, which makes it a period of both opportunity and risk [151]. In animal studies, it is clear that puberty and not age is the agent that increases plasticity [152]. In humans, basal levels of cortisol increase around stage 3 of pubertal development [13]. The amygdala also becomes more reactive to emotional stimuli during pubertal development [153]. There is also evidence that puberty is associated with heightened reactivity to social evaluative stressors [49, 154, 155].

Given all this, puberty may increase the brain’s sensitivity to stress. For example, the amygdala, hippocampus, and striatum, which are all sensitive to stress, increase in volume across adolescence [156]. There is also evidence that GR mRNA in the PFC is significantly elevated in adolescence compared to earlier and later periods of development [22]. At some point in puberty, the stress response becomes sensitive to variations in sex hormones over the course of a woman’s cycle, with females showing smaller responses than males in the luteal phase and comparable responses to males in the follicular phase [157].

If puberty opens a period of heightened plasticity of the brain in response to stressors, does it also have this effect on the HPA axis? Rodent studies suggest this is true. In rats, the HPA axis becomes plastic during the peripubertal period. Specifically, stressors experienced during this period shape a hyper-responsive axis, compared to the same stressor imposed on an adult animal [158]. Relative to adulthood, stressors in adolescent rats were associated with protracted activation of the HPA axis; with an immature negative feedback system, peripubertal rats take longer to mount elevations in glucocorticoids and longer to return to baseline [22]. There is also evidence that stressors in the peripubertal period increase dendritic complexity in the amygdala while decreasing it in the hippocampus [159, 160].

Importantly, sensitive period plasticity cuts both ways. In animal studies and in much of the work on brain plasticity and stress in adolescence, the focus has been on the impact of stressors during this period. But, for individuals who experienced significant adversity early in life but who, during pubertal development, are in more benign conditions, it might be that reactivity of the HPA axis will normalize, becoming more like the reactivity of youth who were reared in benign and/or supportive conditions throughout their lives. This is known as the pubertal stress recalibration hypothesis [1]. Several recent studies support this hypothesis. Children adopted from institutions were studied over several years with nurse exams to assess the pubertal stage and the TSST as a stressor task [10]. An accelerated longitudinal design was used such that at the first assessment, youth ranged from 7 to 14 years and, at last, from 9 to 17 years. Across participants, in the first year, youth adopted from institutions relative to comparison youth showed a blunted cortisol response if they were earlier in pubertal development but a response comparable to comparison youth if they were later in pubertal development [161]. Within participants tracked over the 3 assessments, there was no consistent change in cortisol response for the comparison youth, but for the adopted youth, cortisol reactivity increased to approximate the reactivity of comparison youth [10]. Very similar results were obtained in a cross-sectional study of left-behind children in China [162]. These are children left behind in their rural villages while parents migrate to the cities for work. Typically, they are cared for by older siblings, sometimes just school-age themselves, or by grandparents. Children reunited and living with their parents during puberty showed stress responses to a social evaluative stressor that were comparable to those of children who had not been left behind, while those who were still separated from their parents showed blunted cortisol responses. What is not clear from either of these studies is whether the cortisol response normalizes or is on its way to hyper-responding. This was suggested in a study of children with variations in ELS experiences [163]. Using the CAR, the researchers reported that children at earlier pubertal stages showed a blunted CAR associated with higher ELS scores, while those at later pubertal stages showed a heightened CAR associated with higher ELS scores.

The mechanism(s) involved in recalibration is yet to be determined for the pubertal period; nonetheless, several studies suggest that it may involve changes in the coupling of DHEA and cortisol. In our study of recalibration in previously institutionalized youth, we found that DHEA and cortisol during the TSST were coupled across the pubertal period in comparison to youth. However, for the youth with histories of early institutional care, DHEA and cortisol were uncoupled at early pubertal stages, becoming coupled with pubertal development [164]. Notably, there were no differences in the association of DHEA and puberty as a function of early institutional care. For both groups, DHEA was positively and similarly correlated with the pubertal stage. Thus, what may have been happening is that with increasing DHEA, the system was allowed to recalibrate. This was also the conclusion of a study of cortisol, DHEA, and puberty by King, Graber, Colich, and Gotlib [165].

## 8 SOCIAL REGULATION OF STRESS

Humans are born dependent on adults for survival. Separation or loss of care from adults threatens survival and elicits stress responses in infant monkeys and human children *e.g*., [166, 167]. On the other hand, the presence and availability of the attachment figure is a powerful source of stress buffering for children [168]. This is especially true if the attachment relationship is secure. In addition, it is hypothesized that the security of the attachment relationship and, thus, the efficacy of stress buffering shapes the subsequent reactivity and regulation of the axis to a psychosocial stressor. Of course, in addition to shaping future stress reactivity *via* the efficacy of current social stress buffering, adult caregivers may also affect the child’s capacity to regulate stress through implicit and explicit training in threat appraisal and coping strategies [169].

### 8.1 Animal Models

Social stress buffering has been extensively studied in animals [170, 171]. Removing an infant monkey from its mother, which involves capturing and handling, produces a marked stress response; however, if the infant is immediately returned to the mother, the cortisol response is blocked [168]. Likewise, although squirrel monkeys will allow a surrogate female to carry them if the mother is removed from the group, contact with the “babysitter” does not prevent marked increases in cortisol during the separation period [168]. Although infants tend to focus stress buffering on attachment figures, with development, the presence of other conspecifics can buffer the axis. Thus, among Rhesus macaques, at a year of age when the mother typically leaves last year’s infant to spend several mating days away from the troop, it has been shown in the lab that being with a familiar but not an unfamiliar peer will buffer cortisol responses to several days of maternal separation [172].

As this latter example suggests, species variations in social stress buffering is likely to reflect the social organization of the species and the individual’s age. For example, rat pups cannot see at birth and do not venture from the nest; however, they need to stay close to the mother [171]. For the first two weeks, stimuli from the mother maintain the HPA axis in a relatively quiescent state, and in this state, the infant will approach and not avoid stimuli that produce pain. Because when pain happens in these early days, the stimulus is likely the dam; continuing to approach something that causes pain keeps the pups near the dam even if she steps on them or drops them, for example. Around postnatal day 9, when the pup’s eyes are open, and it begins to explore away from the dam, the HPA axis becomes more active, and corticosterone levels rise. This shifts defensive behavior towards avoidance of threatening stimuli, except when the dam is present. If she is present, corticosterone levels drop, and the pup continues to approach stimuli that produce pain. As the pup matures further, the presence of the dam no longer produces a drop in corticosterone, and the pup moves into a juvenile period of exploration and peer-focused behavior. These shifts in parental stress buffering with development support the developing physical and social needs of the rat pup. In other species, different patterns are observed with development [170].

Animal models paved the way for studies investigating social stress buffering in infants, children, and adolescents. Hostinar and colleagues [171] proposed a developmental model to guide research on mechanisms of social buffering. The working model posited the significance of early social experiences in establishing the biological and psychological systems behind social buffering effects. Guided by this framework, in this section, we will review social buffering development, across infancy, childhood, and adolescence, with a special focus on the significance of attachment relationships.

### 8.2 Infancy

Bowlby [173] theorized that the establishment of the infant’s regulatory systems is dependent, in large part, on the availability and sensitivity of their attachment figures. An internal working model of the likelihood that signals of need will or will not be responded to in a supportive way is established through experiences with caregivers in early life. This internal working model guides future expectations and is hypothesized to influence the adult’s capacity to exhibit buffering effects in the presence of a close social partner.

During the initial 3-4 months, when the HPA axis is highly reactive, sensitive care from parenting figures is associated with better regulation of the HPA axis. Notably, during this time, while undressing and bathing produces marked increases in cortisol in babies, cortisol levels return to baseline faster when parental care is more sensitive [168]. By 8 to 9 months, around the time the baby begins to protest separation from attachment figures and seeks proximity when distressed, the presence and availability of the attachment figure in secure relationships provide a power stress buffer, reducing or preventing a cortisol response to mild stressors [36, 174].

As discussed in the section on sensitive periods, chronic stress in early life has been linked to atypical HPA axis functioning [175]. Inadequate stress buffering in insecure and/or disorganized attachment relationships may have long-term impacts on the reactivity and regulation of the HPA axis. Notably, as discussed elsewhere in this paper, chronic stress in humans is frequently associated with hypocortisolism or the failure to mount a cortisol response to threatening stimuli. To test the hypothesis that insecure attachment in infancy would result in a blunted or hyporesponsive stress system, Fearon and colleagues [175] examined the association of attachment security in infancy with HPA axis reactivity to the TSST in early adolescence in a sample of children growing up in poverty in South Africa. Insecure attachment was associated with a blunted cortisol response to the TSST, especially for males. Notably, for children growing up in the most adverse circumstances, a secure relationship in infancy appears to have buffered the axis, resulting in a more typical cortisol response to the TSST.

The importance of attachment security in buffering stress in early childhood has been noted in naturalistic settings, as well. Toddlers in Germany who were naïve to childcare showed smaller cortisol increases when visiting the childcare setting with their mothers if they were in secure *versus* insecure attachment relationships with the mother [176]. Also, toddlers whose families were below 150% of the federal poverty limit in the US have lower cortisol levels during a well-child medical visit with inoculations if they are in secure *versus* insecure attachment relationships with the parent who is with them [177].

### 8.3 Childhood

In childhood, the attachment relationship remains integral in buffering stress. In an illuminating study, Seltzer *et al*. [178] conducted the Trier Social Stress Test for Children (TSST-C) with girls aged 7 to 12 years. During the recovery period, the girls either recovered with the experimenter present, with the mother present, or with the mother on the phone. Girls whose mothers were physically present, quickly terminated the cortisol response and returned to baseline, while girls with the experimenter present showed the standard response to the TSST-C. Notably, even being able to talk with the mother on the phone reduced the cortisol response and facilitated a more rapid return to baseline, though not as rapid as when the mother was physically present. Being with and talking on the phone with the mother also produced a strong oxytocin response.

The association between attachment security early in life and later HPA axis and oxytocin activity during a non-stress-inducing parent-child interaction has suggested that how attachment security impacts neuroendocrine activity depends, in part, on whether children live in high or low-stress conditions [179]. Stress in this study meant living within range of rockets strikes in Israel. In secure relationships, cortisol decreased across the interaction session if the child lived in control villages that were not subject to rocket attacks, while children from these control villages in insecure relationships showed slight increases in cortisol. Notably, children in secure relationships from high-stress villages showed the highest cortisol that did not decrease and also showed the largest increase in oxytocin. The authors argued that the mode of coping shifted to a form based on affiliation and oxytocin release in the context of high extra-familial stress. While more studies are needed, these findings suggest that the long-term impact of attachment quality on stress responding needs to be studied under a wide variety of conditions calling for attachment regulation of threat during development.

### 8.4 Adolescence

Although emotional connectedness between parents and children remains important throughout life, the potency of parental stress buffering of the HPA axis appears to wane with pubertal development, at least with regards to social evaluative stressors [168]. Specifically, although having the parent help the child prepare for the TSST speech reduces or eliminates cortisol responses during childhood, as puberty progresses, having the parent available during speech preparation is no longer a stress buffer [180]. Importantly, we do not know whether parents continue to buffer the axis when the adolescent faces threats to the physical (*e.g*., pain, surgery) *versus* the social self.

Interestingly, preparing for the TSST with a best friend also does not buffer the HPA response [181]. By emerging adulthood, we know that for males, verbal support from a romantic partner in preparation for the TSST does reduce the cortisol response, while for females having a romantic partner “rub her neck” appears to be effective even though his verbal support is not [182]. For both males and females, recovery from the stressor is accelerated by social support from their romantic partner if the partner provides high *versus* low positive support [183]. Thus, it seems likely that when youth form attachment relationships with romantic partners, neurobiological and endocrine changes that accompany romantic partnering allow that partner to become part of the individual’s stress-regulatory system. When that happens and whether friends can ever serve that function are critical questions for future research. As it is, there is a possibility that in early adolescence, at least, individuals do not have ready access to social partners who can block or buffer cortisol increases to stress, which may be part of why an increase in affective disorders is observed during this period of development.

## 9 EMERGING AREAS

In this section, we identify two emerging research areas in the study of stress physiology and development: (1) racial/ethnic inequalities and discrimination and (2) sleep. The former is viewed as a significant source of stress, while the latter is sensitive to stressors and a key process in stress regulation and resilience. Both research areas are actively being investigated and deserve further attention to optimize the healthy physical and emotional development of children and adolescents.

### 9.1 Racial Discrimination

Racial and ethnic disparities in physical and mental health outcomes are well-documented in the United States [184] and other white-privilege countries (*e.g*., the United Kingdom). Although, racial/ethnic disparities in health outcomes have been studied predominantly among adults, systemic racism has transgenerational effects and indeed is associated with poorer health and well-being in utero *e.g*., [185]. Historical and structural racism continues to impact children's lives throughout development, influencing where they live, the toxins they are exposed to, the availability of quality childcare, and the likelihood that they will be punished more for the same infractions as white children when in childcare or school, family income and material well-being.

As children develop, they become more aware of their social standing and of racial and ethnic discrimination [186]. Because of the heightened vulnerability to social evaluation and threat during adolescence, studies of perceived racial discrimination often focus on the adolescent period [187]. As they enter adolescence, individuals begin to reflect on their racial identity and how their race/ethnicity is perceived by others [184]. Racial discrimination experienced in adolescence is correlated with poorer physical, mental, and academic outcomes, and racial/ethnic disparities in adulthood are thought to be established in adolescence [188]. Neurobiological stress mediating systems, such as the HPA axis, are thought to be some of the pathways through which racial/ethnic disparities and discrimination impact health.

The association between racial/ethnic inequalities and discrimination and stress biology, however, emerges long before adolescence in studies in the US. Racial/ethnic differences in the activity of the HPA axis emerge early in life. Using stressor paradigms, Black infants did not show greater cortisol responses than White infants at 4 months but did at 12 months of age [189]. Chronic cortisol production measured using hair cortisol did not yield race differences among toddlers but did among their mothers [190]. A recent longitudinal study of hair cortisol in Black, Hispanic and White children showed that Black children had higher hair cortisol beginning as early as the preschool years (*i.e*., 2-4 years), and this persisted through middle childhood [191]. Variations in hair cortisol concentrations in that study were correlated with the Child Opportunity Index, especially for the Black children who generally lived in neighborhoods with fewer upward mobility opportunities for children. Fairly consistently, studies examining diurnal cortisol using salivary measures have shown that in middle childhood [192], early adolescence [192], and mid-late adolescence [193], Black children exhibit a flatter diurnal cortisol rhythm than White children. A flatter rhythm, as noted earlier, is associated with poorer physical and mental health. This pattern has been noted among Black adults, as well [194].

Most studies in children that have included other racial/ethnic groups have not noted marked alterations in the adaptation of the HPA axis, either with regard to total cortisol production over time [191], diurnal rhythm [192], or response to psychosocial stressors [195]. However, at least one study of adults noted elevated levels among Hispanic adults relative to White adults, but these elevations were not as great as those noted among Black adults in the United States [196]. Notably, inequalities in income, when controlled statistically, often do not explain or eliminate racial/ethnic differences in stress system functioning [191]. This suggests that other factors associated with race/ethnicity, such as discrimination, may contribute to these differences.

Indeed, alterations in HPA axis functioning have been associated with acute and chronic experiences of racial discrimination, although causality has been difficult to establish [197, 198]. A meta-analysis examining the associations between racial discrimination and HPA axis functioning found, across twenty-seven studies, that the direction of effects was determined by the chronicity of racial discrimination experience(s) [197]. When racial discrimination was measured as an acute event, often through laboratory experiments, the HPA axis appeared more reactive, with more pronounced cortisol output in response to the acute stressors. However, when racial discrimination was measured over longer periods of time and conceptualized as chronic, individuals exhibited a more blunted cortisol response. A similar meta-analysis conducted by Korous, Causadias, and Casper [198] found a small association between racial discrimination and cortisol activity which was moderated by study design; experimental studies examining acute discrimination had significantly larger effect sizes than non-experimental studies investigating racial discrimination *via* surveys and daily diary entries. Results from these meta-analyses speak to the complexity of the relationship between racial discrimination and HPA axis functioning and the need for more nuanced research that addresses both acute and chronic impacts, as well as examining strategies and supports for coping with systemic racism and discrimination.

### 9.2 Sleep

A growing body of research has demonstrated the importance of neurobiological mechanisms in sleep and the bidirectional associations between sleep disturbances and HPA axis activity. Rodent studies have found increases in levels of basal corticosterone and ACTH after sleep deprivation and both acute (1 day) and chronic (8 days) sleep deprivation [199]. The effects of sleep disruption on the HPA axis may also be greater in younger than older rats [200]. Similarly, studies with human adults have shown the effects of sleep deprivation on basal HPA functioning [201], as well as sleep latency and perceptions of sleep quality [202]. Furthermore, alterations in HPA hormones influence sleep patterns. Administration of ACTH in human adults is associated with a reduction of rapid eye movement (REM) sleep, and cortisol administration causes a reduction in both REM and slow wave (SWS) sleep. Administration of CRH leads to lighter sleep [201].

In children, altered HPA functioning has been associated with sleep difficulties. Restricted sleep leads to a diminished CAR [203], and shorter sleep duration is associated with a flatter diurnal slope and lower waking cortisol levels [204, 205]. Lower sleep efficiency is associated with higher afternoon cortisol levels and total daily cortisol production [206, 207]. Furthermore, over-activation of the HPA axis leads to shortened sleep duration, sleep fragmentation, and decreased SWS in children [208].

Focusing on the infancy period, studies assessing sleep quality and HPA axis regulation are limited and have somewhat conflicting results. In one study, at 12 weeks of age, full-term infants who had colic and longer sleep duration had a higher CAR than infants without colic, which suggests an association between developmentally appropriate sleep patterns in infants that may be worsened by effects of colic on cortisol [209]. Poor sleep efficiency and duration, such as more nocturnal awakenings in infancy, lower efficiency in toddlers, and shorter sleep duration in infants and toddlers, have been associated with a flatter diurnal cortisol slope [210, 211]. Importantly, as noted, early life events may be involved in the long-term programming of the HPA axis [212]. Maurer and colleagues [213] compared to sleep in healthy children born very preterm (<32 weeks) and full-term children aged 7-12 years. Very preterm involves increased stress for the child. Children born preterm showed lower levels of cortisol at awakening and lower overall cortisol secretion, lower cortisone in hair, and earlier sleep onset than full-term children. Therefore, this study provides evidence for possible down-regulation of the HPA axis activity and slightly earlier sleep phase in very preterm children during school age. Whether sleep problems play a role in mediating the effect of ELS is an important area for research.

Associations between sleep and HPA regulation may impact children’s mental health. Hatzinger and colleagues [214] conducted a study of preschool-aged children and found that poor sleep patterns were associated with higher HPA axis activity and with behavioral and emotional difficulties. In another study, preschool-aged children with more fragmented sleep had higher awakening cortisol levels, and elevated awakening cortisol levels were associated with teachers’ ratings of internalizing behavior and negative emotionality [211].

Sleep deprivation is an increasing problem during the transition from childhood to adolescence [215]. Indeed, there are changes in sleep duration and quality during the adolescent period, and it is also a time of greater risk for stress-sensitive affective pathology [216, 217]. Both exogenous and endogenous factors may contribute to increasing rates of sleep deprivation. Exogenous factors include earlier school start times, changes in social roles, late-night activities or jobs, and peer influences. Endogenous factors include puberty onset, changes in circadian rhythm, and melatonin levels. These endogenous changes lead both children and rats to shift sleep onsets later and to sleep later into the normal waking period [218]. Tu and colleagues [219] found that peer victimization was more strongly associated with internalizing symptomatology among adolescents with more sleep problems. Further, perceived discrimination was associated with more depressive and externalizing symptoms among adolescents with poorer sleep quality and shorter duration [220, 221]. Finally, Chiang and colleagues [222] found family demands were associated with a smaller CAR among adolescents with longer sleep latency and lower sleep efficiency.

## CONCLUSION

The HPA axis has been the focus of research on stress and development for nearly 70 years. Initially, the research was conducted on animals, especially rats and mice. However, with the development of salivary assays for cortisol, it became feasible to study the reactivity and regulation of this critical neuroendocrine system in humans. The results of an enormous amount of work in both animals and humans has shown that the HPA axis and its regulation undergoes significant developmental changes during ontogeny. There is good evidence of sensitive periods for calibration of the axis early in development and emergent evidence of recalibration during the peripubertal period. The axis comes under strong social regulation during infancy, with attachment figures becoming powerful buffers of the system. There is evidence that puberty reduces the potency of the parent as a social buffer, with little evidence that friends take over the social buffering role, at least until the emergence of romantic attachment relationships in early adulthood. Brain systems involved in activation and regulation of the axis also undergo changes during development, with puberty being an important time for shifting from dependence on the availability of the parent to support regulation of the amygdala by regions in the medial prefrontal cortex to a greater capacity of the individual to regulate their own amygdala reactivity to the threat. Early adversity impacts both the reactivity and regulation of the HPA axis with potentially long-term implications for socioemotional development and health.

The HPA axis is a critical component of the mammalian stress system, but it is not the only system involved in adaptation and resilience in the face of threat and deprivation. As this review makes clear, the HPA system undergoes marked changes with development. One cannot treat the developing axis and thus cortisol measurements during development as if the researcher was measuring the activity of this system in adulthood. A developmental lens is vitally important. This is especially true as the other systems involved in stress responding (*e.g*., immune, oxytocin) also undergo their own changes with development. Thus, understanding how the experience of stress “gets under the skin” to impact human mental and physical health is not simply a matter of measuring the activity of stress-responsive systems. It requires understanding their ontogeny and timing assessments and interpretations to the most relevant developmental period. Even after 70 years of studying this system and asking questions about how early life stress “gets under the skin,” we are only beginning to take a developmental perspective in psycho-endocrine stress research.

## LIST OF ABBREVIATIONS

ACM: Adaptive Calibration Model
AL: Allostatic Load
CAR: Cortisol Awakening Response
CBG: Cortisol Binding Globulin
GRs: Glucocorticoid Receptors
HPA: Hypothalamic-pituitary-adrenocortical
MRs: Mineralocorticoid Receptors
NTS: Nucleus Tractus Solitarius
PVN: Paraventricular Nucleus
SRS: Stress Response System
