# Supplementary material for: The Development of Stress Reactivity and Regulation in Children and Adolescents
Source: Curr Neuropharmacol. 2023 Aug 15;22(3):395–419. doi: 10.2174/1570159X21666230808120504 (PMC10845082; doi:10.2174/1570159X21666230808120504)
Supplement: Supplementary file 1 [file CN-22-395_SD1.pdf]

Supplementary Material

The Development of Stress Reactivity and Regulation in Children and Adolescents

Clarissa Filetti[1,*] and Finola Kane-Grade[1,*] and Megan Gunnar[1]

[1]*Institute of Child Development, University of Minnesota, Minneapolis, USA*

Table S1. Genes with Mutations that Influence the HPA system.

Gene	Chromosome	Function
**Glucocorticoid biosynthesis**		
HSD3B2	1	Codes for the enzyme 3β-HSD that operates in one of the early steps in the synthesis of cortisol. Impairments in this gene results in congenital adrenal hyperplasia.
CYP11B1	8	Codes for the enzyme 11 β-hydroxylase that converts 11-deoxycortisol to cortisol
CYP17A1	10	Codes for an enzyme in the p450 family that is involved in early steps in the formation of all steroid hormones.
CYP11A1	15	Codes for an enzyme in the p450 family that begins the process of converting cholesterol into one of the family of steroid hormones.
PRKAR1A	17	Codes for one subunit of the enzyme Protein Kinase A. Mutations in this gene result in cortisol hypersecretion (e.g., Cushing Syndrome).
MC2R	18	Codes for the protein receptor for ACTH
**HPA axis control**		
LEPR	1	Codes for the protein that makes the leptin receptor. Leptin plays a role in appetite, but it can also inhibit cortisol actions in the PVN of the hypothalamus and in the adrenal.
TBX19	1	Codes for a protein involved in the conversion of POMC to ACTH
POMC	2	Codes for POMC, the prohormone for ACTH
POU1F1	3	Codes for a transcription factor for growth hormone (GH). High growth hormone inhibits ACTH release.
NR3C2	4	Codes for the mineralocorticoid receptor (MR)
NR3C1	5	Codes for the glucocorticoid receptor (GR)
PROP1	5	Mutations in this gene lead to progressive adrenal insufficiency
FKBP5	6	Codes for a co-chaperone of GR that inhibits translocation of GR to the cell nucleus and thus plays a role in glucocorticoid resistance.
CRHR2	7	Codes for the CRH receptor with lower affinity for CRH
LEP	7	Codes for the hormone, Leptin, which regulates appetite, but can also inhibit cortisol actions in the PVN of the hypothalamus and adrenal.
CRH	8	Codes for corticotropin-releasing hormone, the releasing hormone that begins the cascade that results in cortisol production and release.
AVPR1A	12	Codes for the arginine vasopressin receptor. AVP is primarily a posterior pituitary hormone that regulates osmotic balance, but it is also co-expressed in CRH-producing PVN cells where it enhances CRH effects on the production of ACTH in the anterior pituitary.

1875-6190/24 $65.00+.00 **© 2024 Bentham Science Publishers**

CRHR1	17	Codes for the high affinity CRH receptor.
MC4R	18	Codes for melanocortin receptor 4. a-MSH plays a role in regulating cortisol production during the chronic phase of the stress response.
AVP	20	Codes for arginine vasopressin, which when co-produced with CRH in the PVN enhances CRH effects on ACTH release.
**Glucocorticoid metabolism**		
HSD11B1	1	Codes for the protein that catalyzes the conversion of cortisol to cortisone.
H6PD	1	Codes for a protein that is needed for the conversion of cortisol to cortisone.
SRD5A2	2	Codes for the enzyme 5α-reductase 2 that that along with 5α-reductase 1 catalyzes the irreversable conversion of cortisol to dihydrocortisol in the liver. Thus helping to clear cortisol from circulation.
DHRS9	2	Codes for a member of the short-chain dehydrogenases/reductases (SDR) family that play roles in oxireductase activity towards hydrosteroids.
SRD5A1	5	Codes for the enzyme 5α-reductase 1 that that along with 5α-reductase 2 catalyzes the irreversable conversion of cortisol to dihydrocortisol in the liver. Thus helping to clear cortisol from circulation.
CYP3A4	7	Codes for protein that plays a critical role in metabolizing drugs, in addition to its role in the clearance of cortisol in the liver.
AKR1D1	7	Codes for 5β-reductase that in the liver inactivates steroid hormones and plays a crucial role in bile acid synthesis.
SERPINA6	14	Codes for the corticosteroid-binding protein that sequesters cortisol, as only the unbound fraction is bioactive.
ACE	17	Codes for the angiotensin-converting enzyme, SNP mutations of which are associated with larger cortisol responses to stressors.
